# Dependence of the Firearm-Related Homicide Rate on Gun Availability: A Mathematical Analysis

**DOI:** 10.1371/journal.pone.0071606

**Published:** 2013-07-26

**Authors:** Dominik Wodarz, Natalia L. Komarova

**Affiliations:** 1 Department of Ecology and Evolutionary Biology, University of California Irvine, Irvine, California, United States of America; 2 Department of Mathematics, University of California Irvine, Irvine, California, United States of America; University of Adelaide, Australia

## Abstract

In the USA, the relationship between the legal availability of guns and the firearm-related homicide rate has been debated. It has been argued that unrestricted gun availability promotes the occurrence of firearm-induced homicides. It has also been pointed out that gun possession can protect potential victims when attacked. This paper provides a first mathematical analysis of this tradeoff, with the goal to steer the debate towards arguing about assumptions, statistics, and scientific methods. The model is based on a set of clearly defined assumptions, which are supported by available statistical data, and is formulated axiomatically such that results do not depend on arbitrary mathematical expressions. According to this framework, two alternative scenarios can minimize the gun-related homicide rate: a ban of private firearms possession, or a policy allowing the general population to carry guns. Importantly, the model identifies the crucial parameters that determine which policy minimizes the death rate, and thus serves as a guide for the design of future epidemiological studies. The parameters that need to be measured include the fraction of offenders that illegally possess a gun, the degree of protection provided by gun ownership, and the fraction of the population who take up their right to own a gun and carry it when attacked. Limited data available in the literature were used to demonstrate how the model can be parameterized, and this preliminary analysis suggests that a ban of private firearm possession, or possibly a partial reduction in gun availability, might lower the rate of firearm-induced homicides. This, however, should not be seen as a policy recommendation, due to the limited data available to inform and parameterize the model. However, the model clearly defines what needs to be measured, and provides a basis for a scientific discussion about assumptions and data.

## Introduction

Gun violence has been an ongoing problem in the United States of America [1], with an incidence and cost of gun-related homicides that is comparable to some diseases of major public health concern [2]. The rate at which gun-related homicides occur is certainly determined by a complex set of socio-economic and political factors [3], [4]. One of these factors is the degree to which guns are legally available to the general population, determined by the gun policy that is adopted by the country. In the USA, guns can be legally purchased by the general population. However, the relationship between gun availability to the general population and the occurrence of firearm-induced homicides, and thus the effectiveness of gun control policies, has been debated [5], and continues to receive attention. On the one hand, it is argued that gun ownership can protect potential victims when attacked, thus preserving lives [6], [7]. On the other hand, it has been pointed out that lack of control measures enables more people with criminal and violent predispositions to legally obtain guns, thus increasing the rate at which firearm-related attacks and thus homicides occur.

This debate cannot be settled satisfactorily by verbal arguments alone, since these are often driven by opinion, and lack a solid scientific backing. What is under debate is essentially an epidemiological problem: how do different gun control strategies affect the rate at which people become killed by attackers, and how can this rate be minimized? This question can be addressed with mathematical models that describe the interaction between a criminal shooter and one or more people that are the target of the shooter. The gun policy is defined as the fraction of the population that can legally and readily obtain firearms. We aim to analyze the above-described tradeoff and to examine which type of gun policy minimizes firearm-related deaths under different assumptions. Calculations are performed for two scenarios: the assault by a shooter of a single potentially armed victim (what we call a one-on-one attack), or the assault of a crowd of people that can be potentially armed (a one-against-many attack). Note that the former scenario has been documented to be the most prevalent cause of gun-related homicides [8]–[10]. The latter scenario corresponds to incidents such as movie theater or shopping mall shootings and requires a more complicated model. Although such one-against-many attacks are responsible for a small minority of gun-related homicides, they are an important focus of public attention.

The models are based on assumptions that are grounded in and supported by epidemiological data. Yet, it is important to note that a limited amount of such data currently exist, and that future studies will need to confirm these. Because model predictions are a direct consequence of the underlying assumptions, this is necessary to keep in mind. The models give rise to the suggestion that two alternative strategies can minimize the rate of firearm-induced homicides: either a ban of private firearm possession, or a strategy allowing the general population to carry guns. Which strategy minimizes gun-induced homicides depends on the parameters, and here lies the most important contribution of the model: it identifies what needs to be measured statistically in order to improve our understanding about the relationship between legal gun availability and the rate of gun-induced homicides. The important parameters include the fraction of offenders that illegally possess a gun, the degree of protection provided by gun ownership, and the fraction of the population who take up their right to own a gun and carry it when attacked. Limited data exist in the literature that allows a first and preliminary estimate of these parameters, which was performed for illustrative purposes. In the context of these estimates, the model suggests that a ban of private firearm possession, and possibly a certain reduction in the degree of gun availability, might reduce the rate at which firearm-induced homicides occur. However, because these parameter estimates are based on data that were not collected with model parameterization in mind, and because only very limited studies that are relevant currently exist, this model suggestion cannot be interpreted as a solid result that can recommend specific policies. The models represent a first mathematical formulation that examines the relationship between legal gun availability and the rate at which firearm-induced deaths occur. The models give rise to specific predictions, and highlight what needs to be measured to improve understanding. This can serve as a guide for future statistical, epidemiological, and modeling studies.

### Limitations and Interpretation of the Analysis

Because this analysis is very technical in nature and at the same time concerned with a topic that is of interest to a lay audience, it is important to clearly spell out its scope, the inherent limitations, and how results should be interpreted. Any mathematical model of a biological or behavioral process represents by definition a simplification and abstraction of a complex system. A number of assumptions are made about what drives these processes, which are rooted in data that are available in the literature. The model formulates these assumptions in terms of equations, which allow us to take those assumptions to their precise logical conclusions. The results then clearly depend on the assumptions underlying the model, and this is very important to keep in mind when reading this paper, or any paper that deals with mathematical models in biological and behavioral sciences.

In the field of gun violence, it is especially important to be aware of this notion because compared to other fields of biology or epidemiology, only a limited amount of data is available to inform the construction of mathematical models. Therefore, the results reported here should not be viewed as final policy recommendations, but as a first approach to scientifically and logically formulate the issues involved in the gun control debate. One of the aims of this paper is to stimulate a debate about assumptions, statistics, and techniques. By identifying the quantities and parameters that need to be measured, our paper paves the way to further studies which will refine this approach and eventually provide a detailed understanding of how gun availability influences the amount of gun-related and other violence in human populations.

In our opinion, the most valuable yield from our study is a better understanding of what needs to be measured statistically and epidemiologically in order to improve understanding. The models highlight crucial parameters that can determine how gun availability affects the level of firearm-induced homicides, and they suggest what types of statistical data will need to be collected in future research to drive progress. The work performed here can therefore help as a guide for designing statistical studies that are crucially important for a better understanding of these issues, and this would not be possible without designing a mathematical framework. This is a much more important contribution of our work than the actual results that are dependent on assumptions, the nature of which are uncertain to some extent due to the limited amount of data currently available.

To summarize, this paper presents a first mathematical framework to analyze the debate about gun control in the United States, with the aim to steer the debate towards arguing about assumptions and statistics. Equations are used to capture key processes and assumptions that are based on a limited amount of statistical data that are available in the literature. Based on these assumptions, the model gives rise to certain predictions, which are preliminary in nature due to the scarcity of solid statistical studies. The most important contribution, however, is that the model identifies what types of future statistical studies need to be performed in order to improve our understanding of this complex issue.

## Results

### The Modeling Approach

To calculate the effect of different gun control policies on the gun-induced death rate of people, we turn to a mathematical framework that is constructed in this section. This is a new approach, but should be viewed in the context of the larger area of mathematical models that examine aspects of crime, as well as specific shooting scenarios. In the context of crime, a variety of mathematical studies have been performed [11]–[18], although they concentrate on questions that are different from the ones considered here. In the context of shootings in particular, specific scenarios have been investigated, some in the context of war [19]–[22]. Though not directly related to our work, these studies can be viewed as the beginnings of probabilistic and game-theoretic analyses that involve firearms, and thus form an important background for our explorations. At the beginning of the 20th century, Lanchester and Osipov independently developed extensive models of military combat, describing the strength of opposing armies as a function of time, and calculating expected casualties in different situations and in the context of different weapon types [19], [23]. Based on these models, data from specific wars have been used to gain further insights, e.g. [20]. Non-military mathematical analysis of shootings include models of duels and truels, which are generalizations of a duel involving three rather than two participants [21], [22].

We consider the correlates of the total rate (per year, per capita) at which people are killed as a result of shootings. We introduce the variable 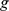 to describe the gun control policy. This quantity denotes the fraction of the population owning a gun. A ban of private firearm possession is described by 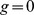, while a “gun availability to all” strategy is given by 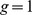. We assume that a certain small fraction of the population is violent (this assumption is relaxed later on by assuming that the population of offenders is not different from the population of victims), and that an encounter with an armed attacker may result in death. The number of offenders that own firearms is a function of the gun control policy 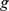 and is denoted by 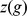; this quantity is proportional to the frequency of gun-related attacks (the proportionality constant does not depend on 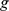 and thus will not alter the results presented here). The probability of a person to die during an attack is also a function of the gun control policy 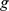 because this determines whether the person and any other people also present at the place of the attack are armed and can defend themselves. This probability is denoted by 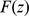. The overall risk of being killed by a violent attacker as a result of shooting is thus proportional to
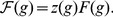
(1)


An important aspect of this model is the form of the dependency of these two quantities on the gun control policy, 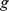. The number of armed attackers, 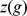, is a growing function of 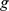, i.e. 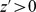. Note, however, that even if offenders are not allowed to legally obtain firearms, there is a probability 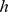 to obtain them illegally. Hence, the value of 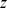 is non-zero for 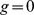. One example of such a function is given by the following linear dependence,
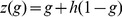
(2)with 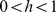. The probability 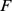 for a regular person to die in an attack (once he or she is at an attack spot) is discussed in the next sections, and depends on the exact situation. A general analysis of expression (1) is presented in the section *Axiomatic modeling*.

### One-against-one Attack

Here we consider the situation where an attacker faces a single individual who can be potentially armed. The fraction of people owning guns in the population is defined by the legal possibility and ease with which guns can be acquired (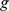), as well as the personal choice to acquire a gun. Moreover, people who own a gun might not necessarily carry the firearm when attacked. Let us denote by 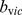 the propensity of potential victims to buy a gun (if legal), and by 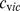 their probability to have it with them at the time of the attack. Then, we can assume that the fraction of people armed with a gun when attacked is given by 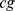, where the parameter 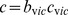 describes the fraction of people who take up their legal right of gun ownership and have the firearm in possession when attacked (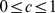). We will model the probability to be shot dead in an attack as
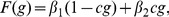
(3)a linear function of 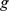, where 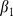 is the probability for an unarmed person to die in an attack, and 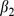 is the probability for an armed person to die in an attack, with 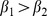. The number of attacks is proportional to 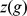 given by equation (2). The aim is to find the value of 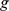 that minimizes the death rate, 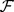, given by equation (1).

#### Optimization results

The first important result is that the killing rate can only be minimized for the extreme strategies 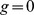 and 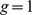, and that intermediate strategies are always suboptimal (this is because 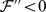 for all 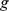). In other words, either a complete ban of private firearm possession, or a “gun availability to all” strategy minimizes gun-induced deaths.

**Figure 1:**
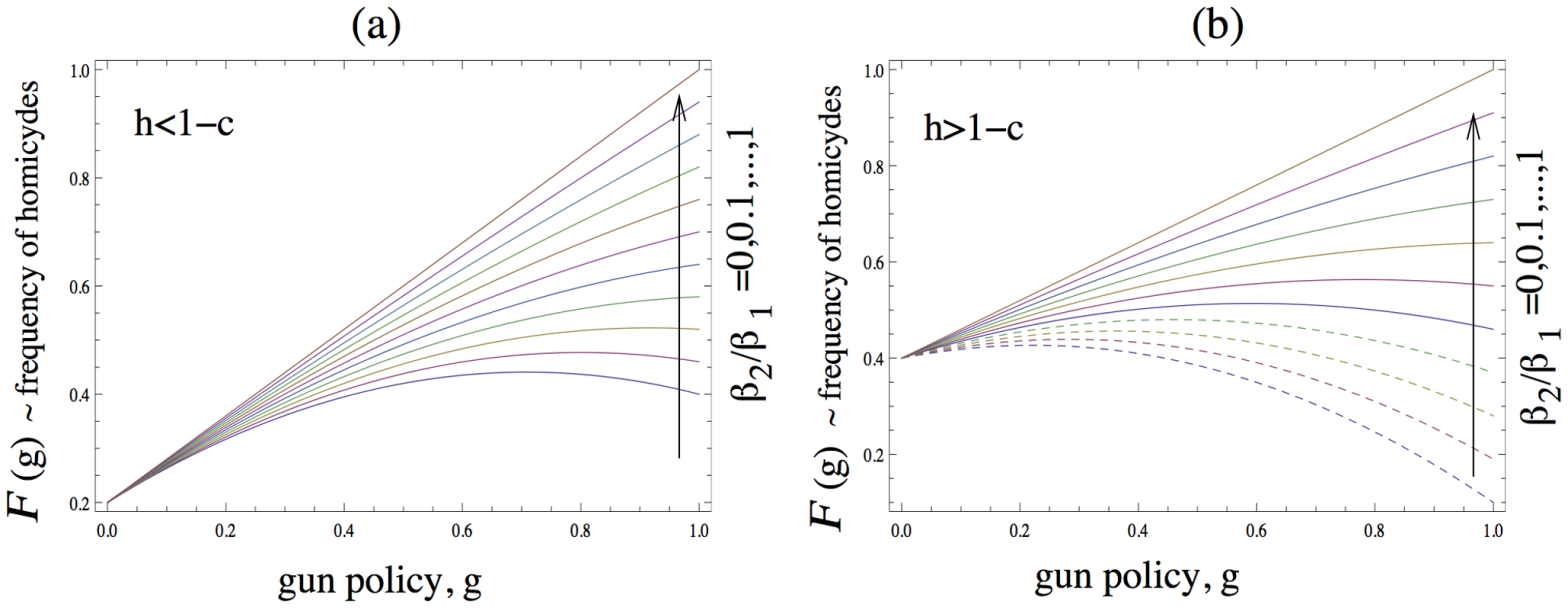
The rate of death caused by shooting in an one-against-one attack, as a function of the gun control policy, 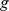, where 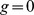 corresponds to a ban of private firearm possession, and 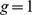 to the “gun availability to all” policy. (a) The fraction of people who possess the gun and have it with them when attacked is relatively low, 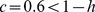 with 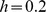. The different lines correspond to different values of 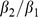. For all values of 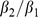, the shooting death rate is minimal for 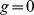. (b) The fraction of people who possess the gun and have it with them when attacked is relatively high, 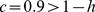 with 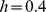. As long as condition (5) holds, the shooting death rate is minimal for 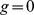 (ban of private firearm possession, solid lines). If condition (5) is violated, then the shooting death rate is minimized for 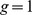 (“gun availability to all”, dashed lines).

Further, we can provide simple conditions on which of the two extreme strategies minimizes death. Let us first assume that
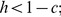
(4)this case is illustrated in figure 1(a) and interpreted below. Now, a ban of private firearm possession always minimizes gun-induced deaths. This can be seen in figure 1(a), where we plot the shooting death rate, 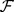, as a function of the gun policy, 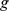, for several values of the quantity 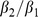. For all of these functions, the minimum is achieved at 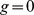.

Next, let us suppose that condition (4) is violated, that is, 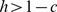, see figure 1(b). In this case, a ban of private firearm possession minimizes death if the following additional condition holds,
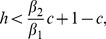
(5)


In the opposite case, the “gun availability to all” strategy minimizes death. Condition (5) defines a threshold value for 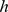, the fraction of offenders that cannot legally obtain a gun but possess one illegally. The threshold value provided by inequality (5) depends on the degree to which gun ownership reduces the probability for the attacked person to die, 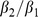 (the smaller the quantity 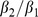, the higher the gun-mediated protection), and on the fraction 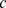 of people who take up their right of legal gun ownership and carry the gun with them when attacked. The right hand side of inequality (5) decays with 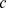. When 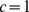 (everybody who has a right to a gun, carries a gun), condition (5) takes a particularly simple form:
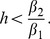
(6)


The case where inequality (4) is violated is illustrated in figure 1(b). Larger values of 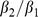 satisfy condition (5), see the solid curves in figure 1(b). For those curves, 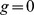 (the total firearms ban) corresponds to the minimum of the shooting death rate. If condition (5) is not satisfied (the dashed curves in the figure), then the 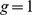 (“gun availability to all”) policy is the optimum.

Note that the key condition (4) relates two quantities, which in some sense are the opposites of each other: The first quantity is 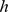, the probability that a potential attacker who cannot legally possess a gun will obtain it illegally and have it at the time of the attack. This can be a measure of law enforcement, with lower values of 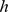 corresponding to stricter law enforcement. The other quantity is 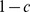, the probability that a person who can legally have a gun will not have it available when attacked. To make the ban of private firearm possession work (that is, to make sure that it is indeed the optimal strategy), one would have to make an effort to enforce the law and fight illegal firearm possession to decrease 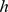. To make the “gun availability to all” policy work, one would have to increase 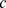, for example by encouraging the general population to have firearms available at all times.

#### Partial restriction of gun-ownership

An important question is as follows. Let us suppose that the total gun ban is impossible due to e.g. constitutional or cultural constraints. Would a partial restriction of gun ownership help reduce the firearm-related homicide rate? It follows that if
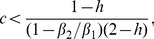
(7)then any decrease in 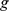 will reduce the gun-related homicide rate. If the value of 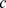 is in the interval



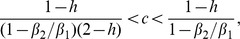
then the maximum death rate corresponds to an intermediate value of 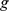 (while the minimum is at 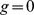, the total ban). This means that if the current state is 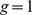, then a partial reduction in 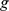 may actually increase the gun-related homicide rate. The reduction must be significant, that is, 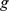 has to be lowered below a threshold, in order to see a decrease in the gun-related death rate. Finally, if



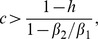
which is the opposite of condition (5), then depending on 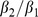, the minimum of 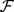 may correspond to 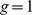. A more general version of condition (7) is given below in section *Axiomatic modeling*.

#### A more general model of the victim population

Equation (3) defines the death probability of a person involved in an attack. Comparing this equation with equation (2) we can see that in this model, we clearly separate the population of attackers and the population of victims. The attackers carry a gun with probability 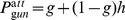, which assumes that the attackers will obtain a gun if it is legally available, and have a probability to also obtain it illegally. The victims carry a gun with probability 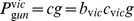, which implies that they never obtain a gun illegally, and even if it is legally available, they may not have a firearm with them at the site of the attack (again, 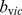 is the probability to buy a gun if legal, and 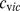 is the probability to have it on them at the time of the attack). This could be an appropriate model for homicide description in suburban and low-crime areas. Below we will refer to this model as the “suburban” model. The separation of the population into attackers and victims is supported by data which document that a large proportion of violent crimes are performed by offenders with a history of prior arrest [24]–[26].

It is, however, possible that the population of victims is similar to the population of the attackers in the context of gun ownership, especially if we model the situation in different socio-economic conditions, such as inner cities. The following model is more appropriate for such situations:
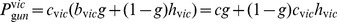



It states that a victim who is entitled to a legal weapon will have the gun available at the time of the attack with probability 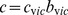. Also, victims who cannot possess a gun legally will acquire it illegally with probability 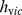 and have it with them with probability 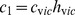. For the attackers, we can formally set
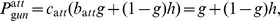
because we assume that 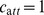 (attackers use up their right to purchase legal weapons) and 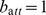 (the attackers carry the weapon with at them at the time of the attack, by definition). This more general model reduces to model (3) if 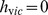 (no illegal gun possession among the victims). If on the other hand we set 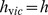, then the population of victims is the same as the population of attackers, apart from the fact that the attackers have a gun with them with certainty (otherwise, there would be no attack), and the victims may not be carrying a gun with them (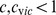). We will refer to this model as the “inner-city” model. In the following text we explore how our conclusions are modified under this more general model.

The 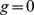 policy is the optimal as long as
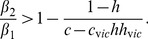
(8)


Note that if 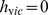, we have 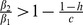, which is the same as condition (5). If 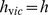 and 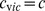, we have 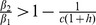, which is a weaker condition than condition (5). In general, increasing 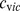 and 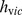 makes condition (8) easier to fulfill. Therefore, we can safely say that if condition (5) is fulfilled for the “suburban” model, then it will be fulfilled for the “inner-city” model.

Furthermore, partial measures to reduce 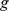 from 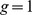 will lead to a decrease in the death toll as long as
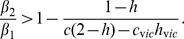



As before, with 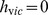 we recover condition (7), and an increase in 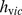 and 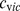 lead to a weaker condition. Again, if partial reduction of the gun ownership improves the death rate in the “suburban” model, it will also work in the “inner-city” model.

### One-against-many Attack

Here, we consider a situation where a shooter attacks a crowd of people, such as in a movie theater or mall shooting. The difference compared to the previous scenario is that multiple people can potentially be armed and contribute to stopping the attacker. In this context, it is worth to point out that the protective effect of gun ownership assumes that the people in the crowd who are being attacked are sufficiently trained in the use of the weapons such that accidental collateral damage does not occur during attempted defense. If this is not the case, this might lower or void the benefits afforded by gun ownership.

We suppose that there are 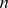 people within the range of a gun shot of the attacker, and 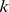 of them are armed. We envisage the following discrete time Markov process. At each time-step, the state of the system is characterized by an ordered triplet of numbers, 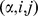, where 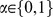 tells us whether the attacker has been shot down (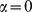) or is alive (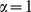), where 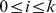 is the number of armed people in the crowd, and 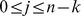 is the number of unarmed people. The initial state is 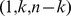.

At each time-step, the attacker shoots at one person in the crowd (with the probability to kill 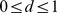), and all the armed people in the crowd try to shoot the attacker, each with the probability to kill 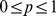. The following transitions are possible from the state 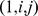 with 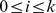, 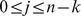 (below we use the convention that expressions of type 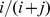 take the value 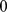 for 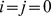):

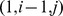
: one armed person is shot, the attacker is not shot, with probability 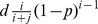;

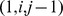
: an unarmed person is shot, the attacker is not shot, with probability 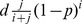;

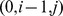
: one armed person is shot, the attacker is shot, with probability 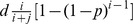;

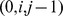
: an unarmed person is shot, the attacker is shot, with probability 
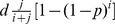
;

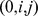
: no potential victims are shot, the attacker is shot, with probability 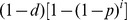;

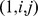
: no one is shot, with probability 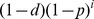.

This model is considered in detail in Text S1, and a *Mathematica* code is provided in File S1, which allows to visualize the results. For 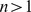, 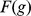 is a decaying function of 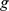, with 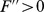 for all 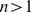. The overall risk of being shot dead in a one-against-many attack can be expressed as the following function:
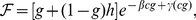
(9)where parameter 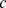 is again the fraction of all the people who take up their legal right of gun ownership and carry the gun with them when attacked. The parameter 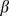 measures the effectiveness of the protection received from the guns (the expression for 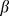 is given in the Methods section), and the function 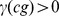 satisfies 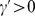, 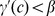.

The optimal strategy that minimizes the gun-induced death rate of people again depends on the degree of law enforcement (i.e. the probability for offenders to obtain firearms illegally). More precisely, we have to evaluate the inequality
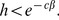
(10)


There are two cases:

- If inequality (10) holds (tight law inforcement and/or gun protection ineffective), then the “ban of private firearm possession” policy (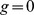) is optimal.
- If 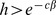 (lax law inforcement and/or gun protection highly effective), then depending on the value of function 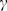 we may have different outcomes, including the the 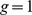 optimum (gun availability to all) or an intermediate optimal policy.

### Axiomatic Modeling

An important aspect of our model is the exact form of the dependency of the quantities 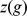 and 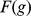 on the gun control policy, 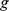. The behavior of the overall risk function, 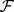 (equation (1)) depends on our assumptions on these two fuctions. Below we discuss some general considerations that apply in different scenarios, and show that most of the conclusions obtained above continue to hold under a more general set of assumptions. We begin by discussion the functions 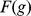 and 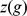.

#### The probability to die in an attack

The probability for a person to die in an attack (once he or she is at an attack spot) is described by the function 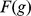. In this paper we consider two different examples of the function 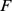. In the case of a one-against-one attack, 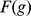 is described by a linear function (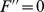). This corresponds to the situation when a shooter attacks a single individual in an isolated setting, i.e. no other people are around to help defend against the attack. It could also correspond to a classroom setting where a shooter attacks the entire class, but only one person (the teacher) can be potentially armed for protection. The most general formula valid for such situation is given by equation (3). In a more complex situation a shooter attacks a group of people, each of which can be potentially armed and contribute to defense. This would correspond to shootings in movie theaters, malls, or other public places. In such a case of a one-against-many attack, the shape of the function 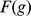 is more complicated, as discussed above. In this paper we show that in the one-against-many case, (1) the function 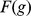 can be nonlinear and (2) under the specific assumptions of our model it has a positive second derivative.

#### The frequency of attacks

The number of armed attackers (and thus the frequency of attacks) is a growing function of 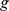, 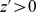. In general and without any further information, there could be three reasonable cases, 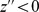, 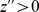, or 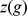 having a sigmoidal shape. There appears to be no reason to assume that the function 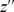 changes sign more than once for 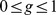. The function 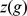 is nonzero at 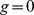 because of the illegal gun market, and we set the value at zero to be 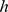: 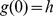. One example of such a function is given by formula (2), which is a linear function of 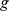. In reality deviations from the linear law are likely for the following reasons: (a) the function 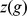 may exhibit a hysteresis-type behavior, that is, the number of attacks may change differently depending on the direction of change of the gun policies; (b) there could be a degree of interaction with 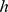, the amount of guns available in the illegal market, as legal guns flood into the illegal market in response to policy changes; (c) spatial inhomogeneity may mean that even if the aggregate number of guns is reduced by reducing 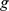, the number of guns in some areas may not decrease, and these are the locations where attackers may be more highly concentrated, meaning a nonlinear (slower) change of 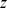 as a function of 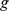. Consider the following argument. The function 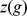 reflects how a change in the general (legal) availability of guns influences the rate of gun attacks. This is assumed to be strongly correlated with the gun availability among the criminals (legal and illegal). If we start from very strict gun control (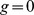), and relax it somewhat by increasing 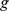 to 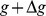, the gun possession among potential criminals will increase by a certain amount (this is the response of the potential criminals to a change in 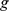 near 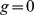). This response has to be compared with the response near 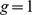 (no gun control). If, starting from a point near 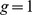, 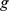 is increased by the same amount 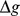, what is the increase in the gun possession among the criminals (this is the response near 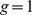)? Presumably, the response near 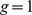 is smaller than that near 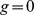, because near 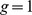 the market is nearly saturated, meaning that most people who want to have a gun will own a gun. On the other hand, near 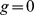 the market is not saturated, and a small change in 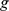 will lead to a relatively large change in gun possession. These considerations inform us that the assumption 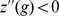 is consistent with reality.

What information can we extract from these general assumptions about the functions 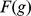 and 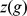
? First of all we note that if both 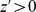 and 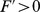, then we have
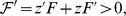
and the minimum of this function is achieved at 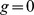. In other words, if more guns in the population increase the frequency of attacks and at the same time increase the danger of being killed in an attack, then the no-guns policy would be the optimal strategy. Moreover, any amount of gun control in this situation would lead to increases safety of the individuals. The assumption 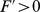 is consistent with the findings of a quantitative study [27], which is discussed later. There is however much debate concerning the protective role of firearms. In order to reflect this, we assume that the function 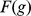 is a decaying function of 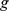, 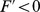, that is, the danger of being killed in an attack decreases with gun availability.

To see how the overall risk of being killed depends on the gun-policy 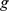, and whether it can have an intermediate minimum or maximum, we consider the second derivative,
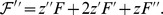



It is easy to see that if both functions 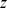 and 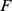 are linear (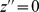 and 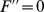), we have 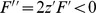, which means that the function 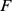 cannot have an intermediate minimum, and the most effective gun control policy must be either 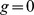 or 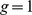.

More generally, if 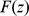 is linear (as it is in the case for the one-on-one attack), and the frequency of attacks is a concave function (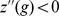, as argued above), we have again,
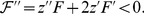
(11)


In other words, as before, the most effective gun policy corresponds to either 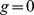 or 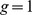. An intermediate minimum in the one-against-one scenario can only happen if 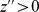, which is arguably not a realistic assumption. To summarize, our results for the global minimum of the total risk function in the one-against-one scenario continue to hold for a more general, nonlinear function 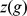 with 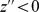.

An intermediate optimum is possible if 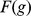 is convex (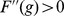, as it is in the case of a one-against-many attack), and if this convexity is sufficiently strong. It follows that if 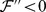 in the case of the linear function 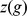, then a nonlinear concave function 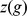 will make this inequality only stronger. In other words, our results on the global minimum of the risk function for the one-against-many scenario continue to hold for a more general, nonlinear function 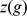.

Can we derive a condition, in this very general setting, which would inform us which one of the two possible optima, 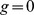 or 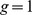, provides the global minimum for the risk function, 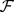
? We have to compare two quantities, 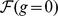 and 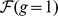. For the one-against-one scenario, we have 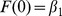 and 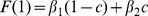. Regarding the function 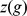, the rate of attacks as a function of 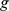, we can set it to 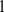 under unrestricted availability of guns, and we further have 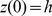, where 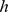 is the fraction of criminals that can (illegally) obtain guns under a complete fire-arm ban. Therefore we can see that conditions (4) and (5) (and all its simplifications) continue to be a valid tool under the most general setting, despite the fact that it was originally derived for a particular, linear model. Further, in the one-against-many scenario, a similar argument shows that condition (10) maintains its validity for a more general class of functions 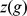.

The next question we address axiomatically is whether a small change in a gun policy starting from 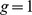 would bring a decrease in the overall rate of gun-related homicides. Some amount of gun control will be beneficial as long as
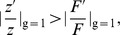
or in terms of the logarithmic derivatives,
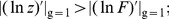
(12)the logarithmic derivatives that appear in the latter inequality have the meaning of the relative rate of change of the functions with respect to 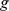. Inequality (12) states that in order for some (partial) gun control measures to be effective, the relative change in the number of gun attacks should be larger than the relative loss in personal safety afforded by the guns. This can potentially be achieved in a variety of ways, for example if gun control measures impact potential attackers more than the general population. Inequality (7) is a particular case of inequality (12), under a linear model for 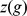. For a one-against-one scenario, where we can use formula (3), we have the following condition: if
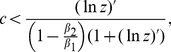
(13)then partial measures (such as reduction in gun ownership) will lead to a decrease in the gun-related homicides.

## Discussion

We analyzed mathematical models in order to calculate the gun-induced homicide rate of people depending on different gun control strategies. In particular, we examined the tradeoff that legal gun availability could either increase the firearm-induced death rates by increasing the number of gun-mediated attack, or reduce the death rate due to protection offered by gun ownership. Such a mathematical framework has so far not been constructed and analyzed, although our work falls into the larger area of shooting and crime modeling, which has been briefly reviewed above.

The gun control strategies in our model were expressed by a parameter that describes the fraction of the population that can legally own firearms. The strategies can range from a ban of private firearm possession to a “gun availability to all” strategy. We first investigated a situation in which one shooter is faced by only a single person that could potentially own a gun and that could fight back against the shooter. This can correspond to a one-on-one attack, such as a robbery, or a school shooting where the only person in the classroom that could carry a gun is the teacher. Subsequently, we examined a different scenario where a shooter faces a crowd of people, all of which could potentially own a gun and fight back against the attacker. This corresponds to shootings in public places such as movie theaters and malls.

In order to understand the implications of these modeling approaches, two aspects need to be considered. First, we discuss to what degree the model formulation is rooted in epidemiological data and how the validity of the model can be tested. Subsequently, we discuss how available statistical data can be used to parameterize the model and to derive specific predictions, based on the model’s assumptions.

### Model Assumptions, Model Testing, and Data

In order to validate the model, data need to be available that inform the functions 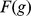 and 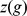 in our model. These are functions that are proportional to the mean rate at which people die during a gun attack, 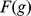, and the mean rate at which gun attacks occur, 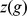. As indicated in the notation, both are assumed to depend on the probability for the general population to own guns, 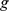, which is determined by the gun policy.

For the one-against-one scenario, the function 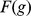 is defined in a straightforward way by 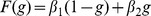. The parameter 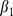 is the probability to die during an attack if the victim does not carry a gun, and the fraction of such victims is by definition 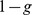. The parameter 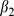 describes the probability for a victim to die during an attack if the victim does carry a gun, and the fraction of such victims is by definition 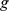. In our arguments, we assume that 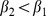, i.e. that gun ownership can offer protection during an attack, because this is one of the central arguments in the current debate. If guns cannot protect against an attack, there is no tradeoff and private firearm possession would confer no benefit to the population. The extent to which gun possession protects against death when attacked is an important parameter that determines the outcome of the model, and is discussed further below in the context of model parameterization. In summary, the formulation of the function 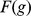 in the one-against-one scenario is robust and not subject to uncertainty.

The formulation of the function 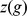 is not straightforward and needs to be backed up by epidemiological data. The most important assumption for our calculations is that increased legal gun availability leads to more attacks by criminals, i.e. 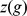 is an increasing function of 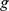. It could potentially be argued that this is not the case because individuals with criminal predispositions can obtain guns illegally, regardless of the gun control policy in place. However, there is some data available in the literature to support our assumption. One study considered two types of populations in California [28]. Among the first group, gun purchases were denied because of a previous felony conviction. In the second group, purchases were approved. Although people in this group had previous felony arrests, they were not convicted. Compared to the group of people who could not obtain a gun, the gun purchasing group was characterized by a significantly higher risk of subsequent offenses that involved a gun. Up to a four-fold difference was observed. This certainly supports the model assumption that 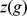 is an increasing function of 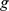, i.e. that the availability of legal gun purchases influences the rate of gun-mediated crimes. Another, related study, found similar results [29], and interestingly further pointed out that while a difference between these two groups was observed with respect to crimes involving guns, no significant difference was observed with respect to crimes that did not involve guns. These results demonstrate that the predisposition to engage in crimes remains the same after gun ownership was denied, and that the prevalence of gun-mediated attack depends significantly on the ability of potentially violent people to obtain guns legally, again supporting the core aspect of our model. In addition to these studies, other papers examined how the incidence of intimate partner homicide depended on the legal availability of guns to potential offenders. When states passed a law that denied guns to abusers under restraining orders, intimate partner homicide rates declined by about 7–12% [30], [31] again supporting the notions described above. Hence, our assumption is quite solidly rooted in available epidemiological data.

One aspect we have no data on is how exactly the function 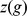 rises with 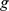. Interestingly, as explored above, axiomatic modeling approaches suggest that our central result continues to hold under all reasonable shapes of this function. More precisely, the optimal policies can be shown to be either a total ban or a guns-for-all policy, as long as the function 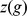 is concave. Moreover, condition (5) holds for any shape of the function 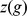. There are, however, aspects of the present model for which additional information about the shape of function 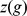 is necessary. Determining the derivative of 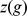 at 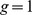 will be very important in the context of partial gun control measures. Quantitative estimates of this derivative (that is, the relative rate of change of 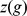) will be crucial for evaluating conditions (12) and (13), which determine whether partial gun control would reduce the rate of gun-related homicides.

The shape of the function 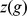, and its derivative in particular, depend on several factors. In the simplest case, one can assume that the fraction of offenders that are not entitled to have a legal gun but get it illegally, 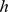, does not depend on 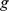, leading to the linear model (2). The opposite assumption however might also be true: as the prevalence of firearms in the general population increases, the fraction of criminals acquiring an illegal gun might increase too. In this case, 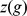 becomes a nonlinear (concave) function of 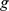. The function 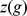 might also be influenced by other processes. The theft of guns by criminals from legal owners could change the effectiveness of gun policies that limit the sales of firearms. It could reduce the potential of the general population to defend itself, while inducing relatively little change in the rate of attack. To some extent, this was addressed in our axiomatic modeling approach, although more detailed studies will be necessary to fully capture the effect of such a phenomenon. It might also be interesting to consider the possibility that without access to assault rifles, attackers resort to the use of explosives. While this might be relevant to the one-against-many type of attack, it is unlikely to play a role in the one-against-one setting.

So far, we have discussed how robust the model assumptions are with respect to available data. With these assumptions in mind, the model gives rise to a set of predictions about the effect of gun availability, 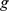, on the rate of firearm-induced homicides, which are discussed further below. It is, however, important to note that the prediction about the dependency of the rate of firearm-induced homicides on the gun availability parameter 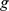 cannot be translated into the expected number of homicides that occur over time in any straightforward way. To draw an analogy, let us consider a hypothetical example about airbag design. We can ask how big the inflated airbag should be in order to minimize the chance of death upon impact. If it becomes too big, it can injure or suffocate the driver. If it inflates too little, it will not protect the driver. Hence, there is some optimum. If we construct a model to calculate the rate at which people get killed during a car crash depending on airbag design, we cannot use this model to fit a time series observed for fatal car accidents. We would have to include other aspects into the model, such as the density of traffic as a function of time, the level of tiredness of drivers or the chance of being intoxicated as a function of time, etc. Similarly, in order to predict gun crime time-series, other aspects would need to be modeled, and it will be those aspects that determine how good the model can fit the data. These are a set of complex factors that go much beyond the scope of our calculations. The occurrence of gun crime as a function of time is complicated, and depends on the time-frame considered [25], [26]. If one looks at this on the time-scale of hours, the number of gun crimes rises during night-time, and falls during the day. If one looks at the time-scale of months, data typically document a clear rise of gun crimes during the summer months. If one looks at the time-scale of years, other, more macroscopic patterns are found, e.g. an overall rise or decline of gun crimes over the years, depending on the exact scenario. Our model was not designed to address these aspects.

### Model Parameterization

One of our results was that either a complete ban of private firearm possession or a policy allowing the general population to carry guns can minimize the rate of firearm-induced homicides, depending on the parameter values. Here, we discuss how the model can be parameterized based on published statistical data, and what such a parameterized model suggests. In this context we point out that any of the published data used to parameterize the model were certainly not designed with our mathematical study in mind and are therefore suboptimal for this purpose. While it is educational and important to attempt model parameterization in this context, the predictions that come out of this exercise are preliminary in nature and await further, more directed statistical studies to refine this work. They should certainly not be viewed as policy recommendations. The power of the mathematical modeling framework, however, is to identify what needs to be measured in future statistical studies to take this research further. It thus serves as a guide that would not exist without the analysis of mathematical models.

An important parameter is the degree of law enforcement relative to the amount of protection that gun ownership offers. If the law is enforced strictly enough, a ban of private firearm possession minimizes the gun-induced death rate of people according to this model.

The question arises how strict the law has to be enforced for the a ban of private firearm possession to minimize the gun-induced death of people. According to our results, this depends on the degree to which gun ownership protects potential victims during an attack and on the fraction of people who take up their legal right of gun ownership and carry the gun with them when attacked. These parameters in turn are likely to vary depending on the scenario of the attack and are discussed as follows.

#### One-against-one scenario

The most prevalent use of guns is a one-against-one scenario and largely involves handguns [8], [9]. For this case, model predictions are relatively simple. Only one of the two extreme strategies can minimize gun-induced deaths, i.e. a ban of private firearm possession or a “gun availability to all” strategy. Intermediate gun policies lead to sub-optimal outcomes. We note that this is not a trivial or necessarily expected outcome. As we have shown in the Axiomatic Modeling section, intermediate optima are in principle possible, although they are observed for unrealistic assumptions about the form of the function 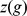. Which strategy minimizes death depends on conditions that are easily interpreted. Gun-induced deaths are always minimized by a firearm ban if 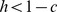. That is, we have to compare 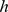, the fraction of offenders that illegally own a gun, with 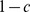, the fraction of the general population that could legally own a firearm but does not have it in possession when attacked. If the condition above is not fulfilled, gun-induced deaths can be minimized by either strategy, depending on the fraction of offenders who illegally obtain firearms relative to the level of protection offered by gun-ownership during an attack. If 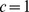 (all people take up their right of gun ownership and carry it when attacked), the conditions is simplest, and a ban of private firearm possession minimizes gun-related deaths if 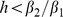, where 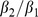 is inversely correlated with the degree of protection offered by gun ownership to a victim during an attack, with 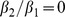 meaning total protection, and 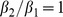 corresponding to no protection associated with gun ownership. All these variables can be estimated from available statistical data, and the implications are discussed as follows.

In order to examine the fraction of offenders that cannot legally obtain a gun but own one illegally, 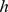, we have to turn to a country with tough gun control laws. If a majority of people can legally own a gun, those that have to obtain one illegally is a negligible fraction. England and Wales have some of the strictest gun control laws since the 1997 Firearms Act, banning private possession of firearms almost entirely with the exception of some special circumstances [32]. Estimating the fraction of potential offenders that illegally carry firearms is a difficult task. Most statistics quantify gun uses during the acts of offense, not among potential offenders. One study tried to fill this gap of knowledge by interviewing a pool of offenders that passed through prison [33]. This was done in the context of the New English and Welsh Arrestee Drug Abuse Monitoring Programme (NEW-ADAM), covering a three year period between 1999–2002, and involving 3,135 interviewees. Among these offenders, 23% indicated that they had illegally possessed a gun at some point in their life. However, only 8% indicated illegal gun ownership within the last 12 months, which we consider a better measure of gun possession associated with committing crimes. More detailed questions revealed that only 21% of people who owned a gun did so for the purpose of an offense. Similarly, among the 8% of people who illegally owned a gun within the previous 12 months, only 23% had taken the gun with them on an offense. Thus, as an estimate for the parameter 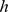, we can say that 23% of the 8% constitutes people who illegally owned a gun which was also present during the offense, and hence 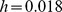.

The fraction of people who legally own a firearm and have it in possession when attacked, 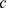, can be partially estimated. Statistical data are available about the fraction of people who personally own a gun in the United States, but no data are available that quantify the probability that these gun owners have the weapon with then when attacked. Approximately 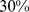 of all adult Americans/households own a gun [34], [35]. Because not all of them will have the firearm with them when attacked, we can say that 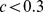. In this scenario, a “gun availability to all” policy can minimize firearm-related deaths if 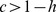, i.e. if 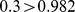. Because this condition is violated, the preliminary parameterization suggests that gun-related deaths might be minimized by a ban of private firearm possession. We would, however, like to stress that further, more extensive statistical data are required for conclusive parameterization, and that this calculation was performed mainly to illustrate how model parameters can be estimated, and how these estimates can be used to determine optimal strategies.

For the sake of the argument, let us consider the extreme scenario where all people who can legally own a gun do so and carry it with them at the time of an attack. This would require an effort by the government to persuade people to purchase firearms and carry them around at all times. As mentioned above, gun-related death is now minimized if 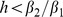. The inverse relative protection that gun ownership provides during an attack (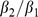) has also been statistically investigated [27]. This is best done in a setting where a large fraction of the general population carries firearms, such as in the USA [1], and this study has been performed in Philadelphia. A total of 677 individuals assaulted with a gun were investigated and the study involved a variety of situations, including long range attacks where the victim did not have a chance to resist, and direct, short range attacks where the victim had a chance to resist. The study found that overall, gun ownership by potential victims did not protect against being fatally shot during an attack. In fact, individuals who carried a gun were more likely to be fatally shot than those who did not carry a gun. This also applies to situations where the armed victims had a chance to resist the attacker, and in this case, carrying a gun increased the chance to die in the attack about 5-fold. The authors provided several reasons for this. Possession of a gun might induce overconfidence in the victim’s ability to fight off the attacker, resulting in a gun fight rather than a retreat. In addition, the element of surprise involved in an attack immediately puts the victim in a disadvantageous position, limiting their ability to gain the upper hand. If the victim produces a gun in this process rather than retreat, this could escalate the attacker’s assault. These data would indicate that 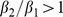, which in turn would mean that a ban of private firearm possession is the only possible strategy that can minimize the gun-related death of people (the probability 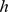 is by definition less than one). The results of this study have, however, been criticized on statistical grounds and it is currently unclear whether 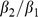 is indeed greater than one [36], [37]. Results are also likely to depend on the geographical location. This study was conducted in a metropolitan area, and results might differ in smaller cities or more rural areas. However, the general notion that gun ownership does not lead to significant protection is also underlined by other studies that discussed the effectiveness of using guns as a defense against attacks [38]–[41], especially in a home setting, although parameter estimates cannot be derived from these studies. In a literature review, no evidence was found that gun ownership in a home significantly reduces the chances of injury or death during an intrusion [41]. On the other hand, this is a very difficult parameter to estimate statistically, because potential shootings that prevent deaths are less likely to be reported to authorities than shootings that do results in death. A precise estimation of this parameter will be a crucial part of future research.

Rather than considering a ban of private firearm possession, it can be more practical to consider the option of partially restricting firearm access. In general, the model suggests that this might either decrease of increase the gun-induced death rate, depending whether the general condition (12) (or its variant for the one-against-one scenario, condition (13)) is fulfilled. Thus, an important message of the calculation is that while a ban of private firearm possession might minimize the death rate, a partial reduction may or may not have this effect, depending on the exact circumstances. Hence, these circumstances need to be studied in detail when designing policies, and our model provides a guide for this. The crucial mathematical condition depends on measurable parameter values, and in particular, on the relative rate of change of the function 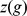, the fraction of attackers that can obtain a gun.

We can see that in general, if 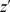 is very small, then condition (12) or (13) will not be satisfied. This is intuitively clear: a small 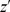 means that as the gun control tightens, the criminals continue to have access to guns through illegal means (thus, a negligible change in 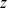). At the same time, if the general population experiences a drop in gun ownership, the total number of gun-related homicides will increase, and such a measure will prove counter-productive. Therefore, to make sure that a partial gun-control measure is effective, it needs to target the right population. An example of such a gun control measure is given by background checks for gun purchasers. In this case, (1) the personal safety of possible victims without the criminal record will not change (no change in 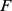); (2) the personal safety of possible victims with criminal record may drop; (3) the incidence of gun attacks will be reduced, as suggested by [28], [29]. Hence, the overall safety of the non-criminal population will increase, and the safety of the population where offenders and victims belong to the same pool will probably change little, because of the two counterbalancing effects of the reduced availability of guns on the victims and offenders (fewer total attacks, fewer guns for defense).

#### One-against-many scenario

Next, we discuss the one-against-many scenario. Here, two different gun control policies can again potentially minimize firearm-induced deaths of people: either a ban of private firearm possession, or arming the general population. However, in the latter case, not necessarily the entire population should carry firearms, but a certain fraction of the population, which is defined by model parameters. As in the one-against-one scenario, which policy minimizes gun-induced fatalities depends on the fraction of offenders that cannot legally obtain a gun but carry one illegally, the degree of gun-induced protection of victims during an attack, and the fraction of people who take up their right of gun ownership and carry the gun with them when attacked. In contrast to the one-against-one scenario, however, this dependence is more complicated here.

**Figure 2:**
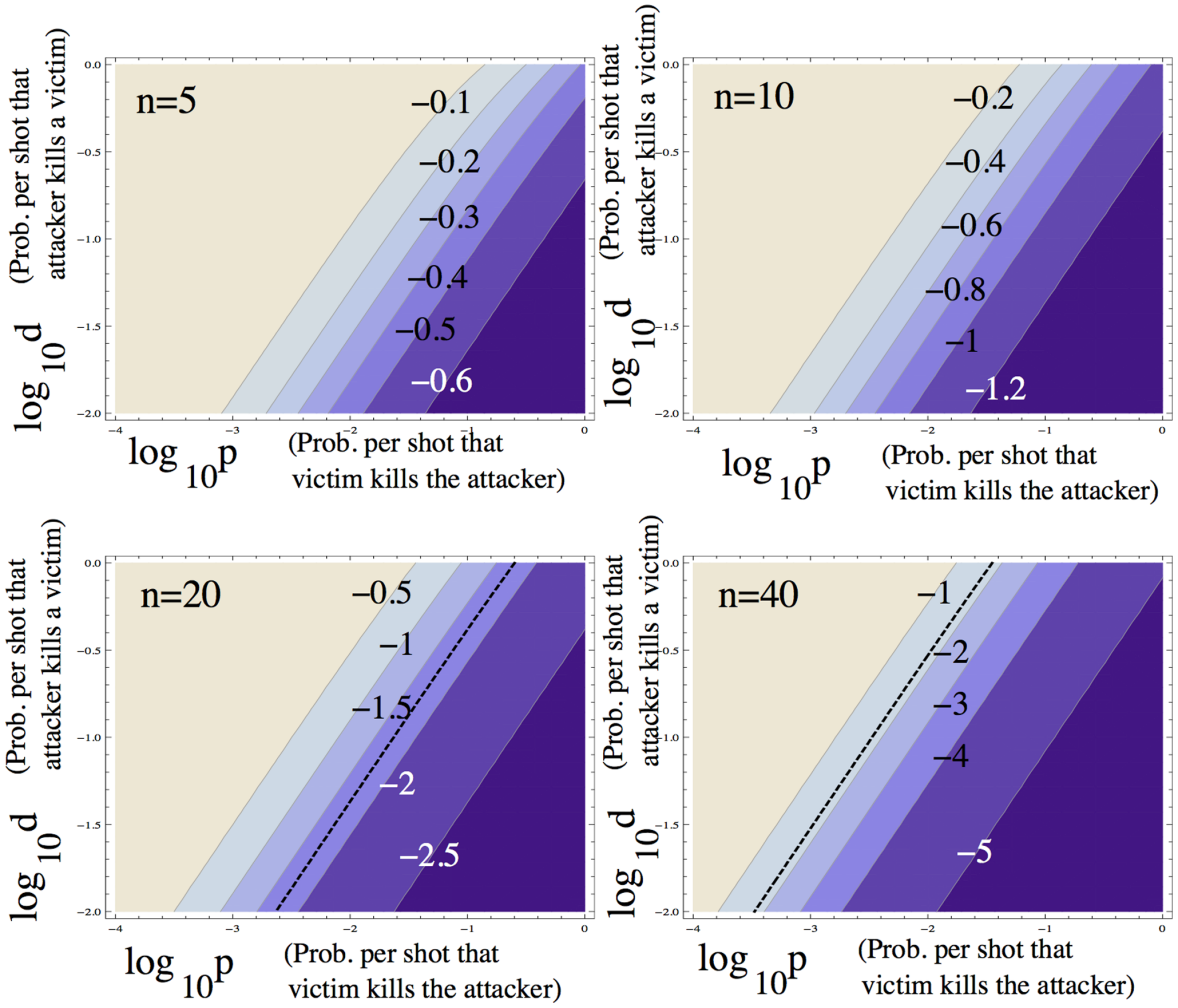
One-against-many attacks: when is a ban of private firearm possession the optimal policy? Presented are the contour-plots of the threshold value 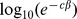 with 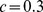, as a function of 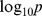 and 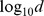 for four different values of 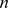. Darker colors indicate smaller values, and the contour values are marked. For each pair of probabilities 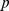 and 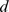, the plots show the highest possible value of 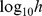 still compatible with the ban of private firearm possession being the optimal policy. The black dashed lines on the bottom two plots indicate the approximate location of the contour corresponding to 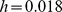; above those lines the ban of private firearm poss ession is the optimal solution. These lines are drawn according to the following relationship between 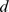 and 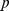: 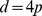 for 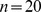, and 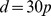 for 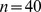. For 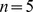 and 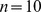, the inequality 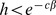 holds for any values of 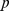 and 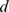, and the ban of private firearm possession is the optimal solution in the whole parameter space.

In this model, the degree of gun-mediated protection against an attack is given by the parameter 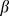. This is a growing function of 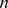 (the number of people involved in the attack) and 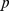 (the probability for a victim to shoot and kill the attacker with one shot). Further, 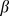 is a decaying function of 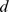, the probability for the attacker to kill a victim with one shot. For a ban of private firearm possession to minimize gun-related deaths, the fraction of violent people that cannot obtain a gun legally but obtain one illegally must lie below the threshold given by condition (10), i.e. 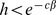. Let us again assume that 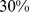 of the general population owns a gun, and for simplicity that they all carry the firearm with them when attacked (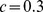). The dependence of the function 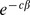 on the parameters 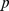, 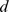, and 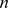 is studied numerically in figure 2. Parameter 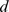, the probability for the attacker to kill a person with one shot, varies between 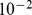 and 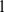. Parameter 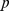, the probability for an armed person to kill the attacker with one shot, varies between 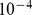 and 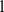. The 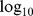 of the right hand side of inequality (10) is represented by the shading, the lighter colors corresponding to higher values. The dependence on parameters 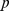 and 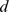 is explored for different numbers of people in the crowd that is being attacked (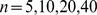). For each case, we ask what values of 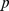 and 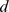 fulfill the inequality 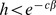, assuming the estimated value 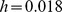. In the parameter regions where this inequality holds, a ban of private firearm possession will minimize deaths, and outside those ranges, it is advisable that a fraction of the population is armed. For 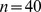 we find that a ban of private firearm possession requires 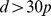. i.e. the attacker needs to be at least 30 times more efficient at killing a victim than a single victim is at killing the attacker. For 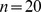 it requires 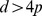. For 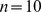 and 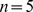, a firearms ban minimizes gun-related deaths for any value of 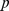 and 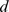. Thus, for smaller crowds, condition (10) is easily satisfied and a ban of private firearm possession would minimize deaths. For larger crowds, it is more likely that arming a certain fraction of the population would be the better strategy because the alternative strategy would require that the attacker is unrealistically more efficient at shooting than the defenders.

The meaning of these numbers further depends on the weapon carried by the attacker. Strictly speaking, the model considered here was designed for attackers and victims with similar weapons. The victims would typically possess hand guns. If the attacker also uses a hand gun, it can be questioned whether the attacker is 30 times more efficient at fatally shooting someone than a victim, even if the attacker is better trained and has more experience. In other situations, more advanced weapons, such as semi-automatic guns can be used to assault crowds, where tens to hundreds of rounds per minute are fired. The victims typically will not possess such powerful weapons. Therefore, their ability to shoot is significantly lower than that of the attacker. We can interpret the results of the model for a situation where the attacker fires a semi-automatic or automatic weapons and the victims respond with hand guns. In this case we must assume that the probability of victims to fire and shoot the killer is significantly (perhaps 2 orders of magnitude) lower than that of the attacker. This shifts the outcome towards a strategy that bans private firearm possession, which would now minimize firearm-related death if a relatively large crowd of 40 people is attacked. For the opposite strategy to minimize death, the attacked crowd would now need to be significantly larger to make up for the increased shooting efficiency displayed by the attacker.

Now, for the sake of the argument assume that 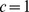, i.e. that everybody who can legally own a gun does so and has it in possession when attacked. We go back to the scenario where the crowd is attacked with a hand-gun and examine the effect. The calculations yield the following results. For 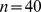, a firearms ban requires that 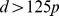, i.e. the attacker needs to be at least 125 more efficient at killing a victim than a single victim is at killing the attacker. For 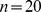, 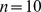, and 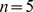, the conditions are 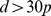, 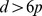, and 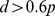. As expected, the assumption that everybody who legally owns a gun carries it when attacked shifts the numbers towards favoring the strategy that involves arming the general population to a certain degree.

Having discussed the one-against-many scenario in some detail, it has to be pointed out that while assaults on crowds generate the most dramatic outcomes (many people shot at once), the great majority of gun-related deaths occur in a one-against-one setting [8]–[10], which generates less attention. Therefore, it is likely that the results from the simpler one-against-one scenario are the ones that should dictate policies when the aim is to minimize the overall gun-related homicides across the country.

### Future Directions of Research

We have discussed the model in the context of what we called the suburban model. That is, we separated attacker and victim populations. We have shown, however, that in the context of the inner city model, the condition for a ban of private firearm possession becomes easier to fulfill. This model does not separate the attacker and victim populations but instead describes a scenario where a large fraction of the population can have criminal tendencies, and where a person may either be an attacker or a victim depending on the situation, e.g. two armed people getting into a fight, drug related crimes, etc.

It also has to be kept in mind that parameter estimates could be different depending on the setting, although there is currently no information available about this in the literature. Related to this issue, it is clear that crime is not uniform with respect to spatial locations. There are areas with adverse socio-economic conditions which are characterized by high homicide rates, and there are areas of relative safety with very few gun crimes. While our model does not take space into account explicitly, it takes into account different scenarios (such as the suburban model or the inner-city model). The optimization problem solved here does not explicitly depend on the spatial distribution of different crime conditions. Further questions about crime management can however be asked if one utilized a spatial extension of this model.

In the present work, two basic scenarios (one-against-one and one-against-many) were discussed. We would like to note that these two scenarios do not include all possible settings in the context of gun violence. There can also be many-on-one or many-on-many cases, and gun violence can also include other important aspects such as accidental deaths, self-injuries, and suicides. Related to this, a blend of the one-against-one, one-against-many, and other scenarios could be considered, although this would introduce further uncertainty due to the necessity to weigh the importance of different scenarios. At present, we preferred to limit our focus to only two scenarios, because the problem is already characterized by a significant amount of complexity.

An issue that we have ignored in our discussion so far are possible deterring effects of gun ownership, i.e. the notion that non-homicide crimes, for example burglaries, could occur less often if those offenders are deterred by the presence of guns in households. Our analysis was concerned with minimizing gun-related homicides, and not crime in general, which is a different topic and should be the subject of future work. If a gun is present in households, and the burglar would consequently carry a gun during the offense, however, the number of gun-related deaths is likely to increase, even if perhaps the total number of burglaries might decrease. This applies especially if guns in the household are unlikely to protect against injury or death, as indicated in the literature [41].

Another link to behavioral decisions which has implications for the influence of gun availability on homicides comes from the punishment literature. It has been shown that if people have the option to punish each other at a cost to themselves, then they use it often illogically as “pre-emptive” strikes [42]. This abuse of costly punishment could be triggered by easy access to firearms. Integrating such behavioral modeling with the framework presented here could be an interesting question to address in future research.

Finally, it is important to note that this paper only takes account of factors related to the gun control policy, and assumes a constant socio-economic background. Of course in the real world a reduction in gun-related (and other) homicides would require improvement of the living and work conditions and education of underprivileged populations. Here we do not consider these issues. It is important to emphasize that cultural and socio-economic differences exist between different countries, between cities or regions within the same country, and even between different time periods within the same location, making it difficult to draw direct comparisons. A lower or higher rate of gun-related deaths is not only a function of gun control policies, but also of those other factors. Comparisons in the context of this model are only possible within the same cultural and socio-economic space. We single out the direct effects of gun-control policies and investigate those under fixed cultural and socio-economic circumstances. Therefore, at present, there is no straightforward way to relate our results to many statistical studies that compare gun violence in different cities, countries, or over time following changes in gun policies [43], [44].

Related to this, it has been reported that homicide growth rates show the same form for different city sizes in a study quantifying aspects of murders in Brazilian cities for a defined period of time [45]. Similarly, it has been pointed out that processes relating to economic development are quite general and shared across cities, nations and different times, and many diverse properties of cities have been shown to be power law functions of population size [46]. At the present stage, however, it is not clear how this can apply to the current model. For example, both the UK and Switzerland show drastically lower rates of firearm-induced homicides than the US. Private firearm possession is largely banned in the UK, while gun prevalence in Switzerland is wide-spread. Extensive safety training is required for individuals possessing guns in Switzerland and the prevalence of wealth and poverty is significantly different from the US or the UK, making comparisons across different socioeconomic backgrounds very challenging. Future research may shed light onto ways of making the current model more “universal”, enabling to draw comparisons among different populations.

### Conclusions

This paper provides the first mathematical formulation to analyze the tradeoff in the relationship between legal gun availability and the rate of firearm-induced death: while more wide-spread legal gun availability can increase the number of gun-mediated attacks and thus the firearm-induced death rate, gun ownership might also protect potential victims when attacked by an armed offender, and thus reduce the firearm-induced death rate. The main contributions of this study are as follows. (1) We created a mathematical model which takes account of several factors that are often discussed in the context of gun-induced homicide. The model is based on a set of assumptions that are supported by previously published empirical data, as was discussed in detail. For assumptions where no epidemiological data were available for model grounding, we employed axiomatic modeling approaches which showed that most model properties remained robust within epidemiologically reasonable constraints. The model suggests that the rate of firearm-induced homicides can be minimized either by a ban of private firearm possession, or by the legal availability of guns for everyone, depending on the parameter values. While there is strong indication that the model assumptions and hence the properties are consistent with data, it will be important to collect more data to back up the underlying assumptions more strongly. (2) We illustrated how model parameterization can be useful in deciding the correct strategy to minimize the rate of gun-induced homicide by attempting parameter estimations, based on data that are available in the literature. These data have not been collected with this purpose in mind and are certainly not extensive enough to allow conclusive parameter estimates. With this constrain in mind, the preliminary parameterization of the model suggests that the firearm-induced homicide rate might be minimized by a ban of private firearm possession, and possibly reduced if gun availability is restricted to a certain extent. Due to the preliminary nature of the data used for model parameterization, however, this should not be viewed as a policy recommendation, which will require detailed epidemiological studies that collect extensive data sets specifically geared towards parameterizing the model. (3) Possibly the most important contribution of our study is as follows. Our model is based on several variables/parameters, and it shows in what way these parameters may contribute to the delicate balance of factors responsible for the prevalence of gun-related homicides. To improve understanding, these crucial parameters need to be measured by epidemiological and statistical studies. The model identifies these parameters and can thus serve as a guide for the design of these studies. Our work will hopefully stimulate such empirical studies, and also steer the debate about gun control towards a scientific approach where assumptions, data, and methodologies are discussed.

## Analysis

Detailed analysis of the stochastic models used in this paper is provided in Text S1.

## Supporting Information

Text S1 **This text provides details of the mathematical analysis of the one-against-many model.**
(PDF)

File S1 **A** ***mathematica*** **file which allows to visualize the results of modeling in the context of the one-against-many model.**
(NB) Click here for additional data file.
