# Supplementary material for: Dependence of the Firearm-Related Homicide Rate on Gun Availability: A Mathematical Analysis
Source: PLoS One. 2013 Jul 26;8(7):e71606. doi: 10.1371/journal.pone.0071606 (PMC3724776; doi:10.1371/journal.pone.0071606)
Supplement: Text S1 — This text provides details of the mathematical analysis of the one-against-many model. (PDF) [file pone.0071606.s001.pdf]

Dependence of the firearm-related homicide rate on gun availability: a mathematical analysis

Dominik Wodarz and Natalia L. Komarova

Text S1

Here we provide details of the stochastic model used in the main paper in the context of the "one-against-many" scenario.

The stochastic process. The absorbing states of the stochastic system are (i) all the states $(0, i, j)$, where the attacker has been shot, and (ii) $(1, 0, 0)$, where all the people have been shot. The state $(0, 0, m)$ is unreachable from $(1, i, j)$. Let us denote by $h_{i,j\to l,m}$ the probability to be absorbed in state $(0, l, m)$ starting from state $(1, i, j)$. The goal is to calculate the function $F(g)$, which is proportional to the probability for an individual involved in an attack to die.

We have $h_{i,j\to l,m} = 0$ as long as $l > i$ or $m > j$. Further, $h_{i,j\to 0,m} = 0$ for all (i, j, m). Let us denote by $\lambda_{i,j}$ the probability to be absorbed in state $(1, 0, 0)$ starting from state $(1, i, j)$. We must have

$$\sum_{l=1}^{i}\sum_{m=0}^{j} h_{i,j\to l,m} + \lambda_{i,j} = 1. \tag{1}$$

The following equations can be written down for these variables, by using a one-step analysis:

$$\begin{aligned}
h_{i,j\to i,j} &= (1-d)[1-(1-p)^i] + (1-d)(1-p)^i h_{i,j\to i,j},\\
h_{i,j\to i-1,j} &= \frac{di}{i+j}[1-(1-p)^{i-1}] + \frac{di}{i+j}(1-p)^{i-1}h_{i-1,j\to i-1,j} + (1-d)(1-p)^i h_{i,j\to i-1,j},\\
h_{i,j\to i,j-1} &= \frac{dj}{i+j}[1-(1-p)^{i}] + \frac{dj}{i+j}(1-p)^{i}h_{i,j-1\to i,j-1} + (1-d)(1-p)^i h_{i,j\to i,j-1},\\
h_{i,j\to l,j} &= \frac{di}{i+j}(1-p)^{i-1}h_{i-1,j\to l,j} + (1-d)(1-p)^i h_{i,j\to l,j}, \quad l < i-1,\\
h_{i,j\to i,m} &= \frac{dj}{i+j}(1-p)^{i}h_{i,j-1\to i,m} + (1-d)(1-p)^i h_{i,j\to i,m}, \quad m < j-1,\\
h_{i,j\to l,m} &= \frac{di}{i+j}(1-p)^{i-1}h_{i-1,j\to l,m} + \frac{dj}{i+j}(1-p)^{i}h_{i,j-1\to l,m} + (1-d)(1-p)^i h_{i,j\to l,m},\\
&\quad l < i, \quad m < j.
\end{aligned}$$

The values for all $h_{ij\to lm}$ can be calculated recursively from this system. For completeness, we also write down the equation for the variable $\lambda_{i,j}$:

$$\lambda_{i,j} = \frac{di}{i+j}(1-p)^{i-1}\lambda_{i-1,j} + \frac{dj}{i+j}(1-p)^{i}\lambda_{i,j-1} + (1-d)(1-p)^i\lambda_{i,j}.$$

One can check that equation (1) holds.

Given the probability to carry a gun cg, the probability to have i individuals out of n armed is given by

$$\frac{n!}{i!(n-i)!}(cg)^i(1-cg)^{n-i},$$

and the probability to have l armed and m unarmed individuals still alive after an attack is given by

$$\sum_{i=1}^{n}\frac{n!}{i!(n-i)!}(cg)^i(1-cg)^{n-i}h_{i,n-i\to l,m}.$$

Therefore, the probability to have k people out of n to survive the attack is

$$P_k=\sum_{l=1}^{k}\sum_{i=1}^{n}\frac{n!}{i!(n-i)!}(cg)^i(1-cg)^{n-i}h_{i,n-i\to l,k-l}.$$

Examples of this probability distribution for three different values of g are shown in figure S1(a). The expected number of people that survive is given by

$$\sum_{k=1}^{n}P_k k.$$

Therefore, the function $F(g)$, proportional to the probability to be killed in an attack, is given by

$$F(g)=1-\frac{1}{n}\sum_{k=1}^{n}P_k k. \tag{2}$$

The case $n=1$. First of all, we can apply the one-against-many attack model for the case $n=1$. As expected, $F(g)$ for $n=1$ is a linear function of g, which can be written as

$$F(g)=\beta_1(1-cg)+\beta_2 cg,$$

with parameters β_1 and β_2 giving rise to the threshold value of h,

$$\frac{\beta_2}{\beta_1}=\frac{d}{d(1-p)+p}.$$

We can see that the stochastic model informs our previous simple model by relating the quantity β_2/β_1 to the probability of the attacker to kill a victim with one shot, d, and the probability of a victim to shoot the attacker, p. As expected, the quantity β_2/β_1 grows with d and decays with p. In other words, the gun-mediated protection decays with d and it grows with p.

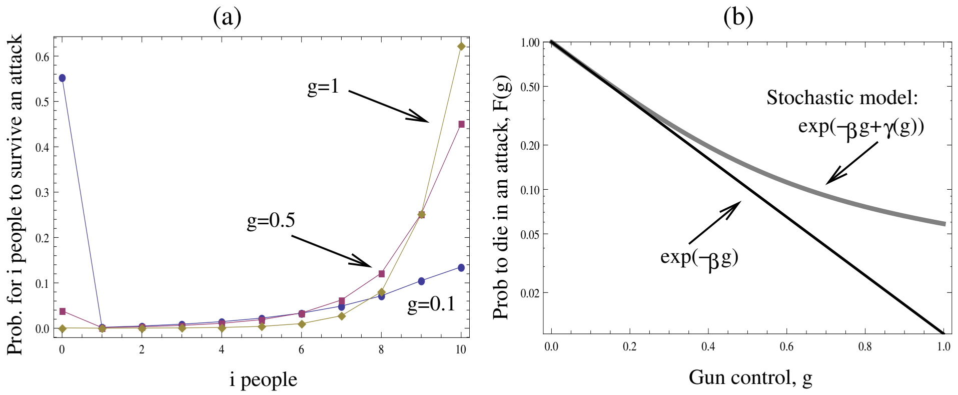

Figure S1: **Results of the stochastic model of the one-against-many attack.** (a) A typical shape of the probability distribution of the number of people who survive an attack, plotted for three different values of g. (b) The probability to survive an attack, $F(g)$, as a function of the gun control (please note the logarithmic scale). The function $F(g)$ obtained in formula (2) is plotted by a thick gray line. The solid black line corresponds to approximation $e^{-\beta g}$ with β given by formula (3). Other parameters are $n = 10$, $d = 0.1$, $p = 0.02$, and $c = 1$.

The shape of the function $F(g)$. The expression for F for $n > 1$ is complicated. A *Mathematica* code is provided in Supplement S2 which evaluates it for any given set of parameters. A typical shape of this function is shown in figure S1(b), the thick gray line. We can calculate the approximation for this function for small values of g, by setting

$$F(g) \approx 1 - \beta c g,$$

where

$$\begin{aligned}
\beta &= \frac{p}{n(d(1-p)+p)^n}\left(\sum_{j=1}^{n-1} j^2 d^{n-j}(1-p)^{n-j-1}(d(1-p)+p)^{j-1}\right. \\
&+ \left. n^2(1-d)(d(1-p)+p)^{n-1}\right) \\
&= \frac{1}{np^2}\left(2d^2 + dp - 2d^2p - 2dnp + n^2p^2\right. \\
&- \left.(2d(1-p)+p)d^{n+1}(1-p)^n(d(1-p)+p)^{-n}\right). \qquad (3)
\end{aligned}$$

Instead of working with the particular model described above, let us design a simpler model, which would retain some of the properties of the stochastic model considered, but be easier to analyze. First we notice that $F' < 0$ and

$F'' > 0$. Consider the following approximation of this function which satisfies $F' < 0$ and $F'' > 0$:

$$F(g) \approx e^{-\beta c g}. \quad (4)$$

Figure S1(b) shows that while expression (4) is a good approximation of the function $F(g)$ for small values of g, it deviates from the function F as g approaches 1, see the solid black line. The function F given by exact formula (2) has a higher curvature for larger values of g (the thick gray line in figure S1(b)), so the actual expression for F is given by

$$F(g) = e^{-\beta c g + \gamma(cg)}, \quad (5)$$

where $\beta > 0$ and $\gamma'(c) < \beta$, such that $F' < 0$ for $0 \leq g \leq 1$. Approximation (4) is shown in figure S1(b), the black line. In equation (5), β is given by expression (3).

If $\gamma = 0$, we have $\mathcal{F}'' < 0$, and the optimal strategies are the same as in the one-against-one attack: only the two extreme strategies can minimize the gun-induced death rate of people, i.e. either a "ban of private firearm possession" strategy ($g = 1$) or the "gun availability to all" strategy ($g = 1$). Conditions $h < e^{-c\beta}$ (or $h < e^{-\beta}$ if $c = 1$, see the main text) help separate the two cases. If we take account of the existence of a nonzero correction $\gamma > 0$ in the expression for $F(g)$, it follows that the above inequalities still plays a key role in separating two different cases, as described in the main text.

Different types of weapons. In the main text we use the above stochastic model to study the one-against-many type of attacks. We consider two situations: (1) The attacker has the same type of weapons as the potential victims (e.g. handguns); (2) The attacker has a more powerful weapon, i.e. a machine gun. The latter situation can be modeled in two ways. One could assume that the time-intervals between shots are different for hand-gun users and machine-gun users. Alternatively, one can keep the time-intervals the same, and assume that during one time-interval, a machine-gun user has several (many) attempts to aim at the victims, thus increasing his probability to kill. In this paper we adopted the second approach and asumed that the probability of a successful shot by the attacker is significantly larger than that for the victims ($d \gg p$). Two two approaches become identical in the continuous limit.
